# Kynurenine pathway activation following moderate-to-severe traumatic brain injury with hemorrhagic shock and the effects of aeromedical evacuation

**DOI:** 10.1016/j.bbih.2026.101389

**Published:** 2026-10-13

**Authors:** Harm J. van der Horn, Kristin E. Connors, Carissa J. Mehos Milliken, Upasana Nathaniel, Koen Visser, Tracey V. Wick, Samuel D. Miller, Andrew P. Gigliotti, Timothy B. Meier, Sophie Erhardt, Rachel Kinsler, Claude van der Ley, Martijn van Faassen, Ido P. Kema, Joukje van der Naalt, Rebekah Mannix, Andrew R. Mayer

**Affiliations:** a Neurology Department, University of Groningen, University Medical Center Groningen, Groningen, the Netherlands; b The Mind Research Network/Lovelace Biomedical Research Institute, Pete & Nancy Domenici Hall, 1101 Yale Blvd. NE, Albuquerque, NM, 87106, USA; c Department of Neuroscience, University of New Mexico School of Medicine, Albuquerque, NM, 8713, USA; d Department of Neurosurgery, Medical College of Wisconsin, Milwaukee, WI, USA; e Department of Cell Biology, Neurobiology and Anatomy, Medical College of Wisconsin, Milwaukee, WI, USA; f Department of Biomedical Engineering, Medical College of Wisconsin, Milwaukee, WI, USA; g Department of Physiology & Pharmacology, Karolinska Institutet, Stockholm, 171 77, Sweden; h U.S. Army Aeromedical Research Laboratory, Fort Rucker, AL, USA; i Department of Laboratory Medicine, University of Groningen, University Medical Center Groningen, Groningen, the Netherlands; j Boston Children's Hospital, Boston, MA, 02115, USA; k Neurology Department, University of New Mexico School of Medicine, Albuquerque, NM, 87131, USA; l Psychiatry Department, University of New Mexico School of Medicine, Albuquerque, NM, 87131, USA; m Psychology Department, University of New Mexico, Albuquerque, NM, 87131, USA

**Keywords:** Kynurenine, Inflammation, Tryptophan, Polytrauma, Large-animal model

## Abstract

While moderate-to-severe traumatic brain injury (msTBI) frequently occurs alongside extracranial injury, the pathophysiology of polytrauma remains incompletely understood. Aeromedical evacuation (AE) from remote settings may introduce additional physiological stress. The kynurenine pathway (KP) is of interest due to its involvement in inflammation, excitotoxicity, oxidative stress, and energy metabolism.

This study used a porcine model to examine serum KP metabolite concentrations following closed-head rotational msTBI with 40% hemorrhagic shock (TBI + HS) and the effects of AE. Yucatan minipigs (n = 24) were randomized to Mock flight (stationary at ground level at 5400 ft), standard (∼8500 ft), or tactical evacuation (∼500 ft above ground level) using a U.S. Army Black Hawk helicopter. Blood samples were collected at baseline, 35, 105, and 210 min, and 24 h post-injury. Serum kynurenic acid (KYNA), quinolinic acid (QUIN), and 3-Hydroxykynurenine (3-HK) concentrations were quantified. A separate sham cohort (n = 10) was analyzed at baseline, 35 min, and 24 h. For TBI + HS, microglial activation was inferred from ionized calcium-binding adaptor molecule 1 (Iba1) immunohistochemistry in post-mortem brain tissue at 24 h. Results showed markedly increased KYNA, QUIN and KYNA/QUIN ratio following TBI + HS, peaking at 35 min post-injury and returning to baseline or below by 24 h. 3-HK was elevated at baseline and showed only a modest increase at 210 min. Robust increases in KP metabolite concentrations were observed at 35 min relative to sham. Both AE conditions were associated with lower 24-h KYNA compared with Mock flight. No differences in microglial activation, as assessed by Iba1 staining, were observed between AE groups. However, within the TBI + HS cohort, strong negative correlations were observed between KYNA and KYNA/QUIN (at 35-210 min) and microglial activation. KYNA and the KYNA/QUIN ratio were positively associated with lactate, glial fibrillary acidic protein, and ubiquitin carboxy-terminal hydrolase L1. These findings indicate robust KP activation following TBI + HS, likely caused by the acute release of catecholamines and inflammatory markers, with minimal effects of AE. The mechanistic role of the KP in polytrauma needs to be further assessed in clinical populations to evaluate its potential for targeted modulation, for example focused on an immunomodulatory role for KYNA.

## Highlights

• Kynurenines were examined in a porcine polytrauma model (TBI + hemorrhagic shock).
• Polytrauma caused acute increases in blood kynurenic and quinolinic acid.
• Helicopter evacuation lowered kynurenic acid at 24 h relative to mock flight.
• Higher acute kynurenic acid levels associated with less microglial staining at 24 h.
• Associations were found with blood markers of metabolic stress (e.g., lactate).

## 1 Introduction

The kynurenine pathway (KP) is the major degradation route for tryptophan (TRP) and plays a role in the pathophysiology of many diseases (Badawy, 2017; Pocivavsek et al., 2024; Schwarcz et al., 2012)**,** including traumatic brain injury (TBI (Meier and Savitz, 2022)). Activation of the KP occurs under acute catecholamine-mediated stress and inflammatory conditions through a rapid increase in TRP flux via the enzymes indoleamine 2,3-dioxygenase (IDO1) and tryptophan 2,3-dioxygenase (TDO2) (Badawy and Evans, 1976; Foley et al., 2021; Höglund et al., 2019; Rittig et al., 2016), followed by transcriptional induction of these enzymes over a longer time frame during the glucocorticoid-mediated stress response (peaking around 3-8 h post-glucocorticoid release (Altar et al., 1983; Danesch et al., 1983; Nakamura et al., 1987)) and inflammation (peaking at 8-48 h post cytokine release (Ozaki et al., 1987; Pfefferkorn et al., 1986)). This results in increased production of kynurenine (KYN), which can subsequently be metabolized into downstream metabolites including kynurenic acid (KYNA) and quinolinic acid (QUIN) (Badawy, 2017; Pocivavsek et al., 2024). These metabolites are neuroactive, with KYNA acting as a *N*-methyl-D-aspartate (NMDA) receptor antagonist that is generally considered neuroprotective, and QUIN, an NMDA agonist, becoming neurotoxic at elevated concentrations. Thus, a higher KYNA-to-QUIN ratio can be considered neuroprotective. Importantly, under physiological conditions QUIN plays a role in cellular energy metabolism as a precursor in the de novo synthesis of nicotinamide adenine dinucleotide (NAD^+^), an essential cofactor for ATP production (Braidy et al., 2011; Liu et al., 2007). Another key intermediate metabolite is 3-hydroxykynurenine (3-HK), which possesses important redox-active properties and can exert both antioxidant (Goda et al., 1999) and pro-oxidant (Okuda et al., 1998) effects. Accumulation of 3-HK under disease conditions (time frame of hours to days (Skouras et al., 2016)) may contribute to oxidative stress and tissue damage.

A previous study in humans with severe TBI has shown that cerebrospinal fluid levels of QUIN and KYNA are significantly increased in the (post-)acute phase, with higher QUIN levels associated with poorer outcome (Yan et al., 2015). In athletes with histories of concussion, elevated QUIN and 3-HK levels were associated with structural brain abnormalities (Meier et al., 2016, 2025). Human studies on polytrauma have demonstrated elevated serum KYN relative to TRP in the first days to weeks following injury (Lögters et al., 2009; Ploder et al., 2009), indicative of increased IDO1 activity, particularly in non-survivors (Ploder et al., 2009). Also, in a porcine model of hemorrhagic shock (HS) combined with tissue injury (bilateral comminuted femur fractures), plasma KYN was also found to be increased within the first hours after injury (Lacroix et al., 2023). To our knowledge, no study has specifically characterized the KP after severe polytrauma with concomitant TBI. This represents an important knowledge gap as intra- and extracranial injuries frequently co-occur in major trauma, especially in combat situations, and may exert reciprocal and potentially opposing effects on systemic and cerebral pathophysiology (Kumar et al., 2015; Maas et al., 2022). For example, hypotension is a critical factor following TBI, arising from HS or neurogenic mechanisms related to the injured brain, and is associated with increased mortality and worse outcomes (Lee et al., 2024; Mayer et al., 2019; McHugh et al., 2007). A more detailed understanding of the underlying biochemical mechanisms of these injuries may help identify potential targets for acute therapeutic intervention and optimize evacuation strategies in case of combat situations. The latter is relevant, as hypobaria associated with aeromedical evacuation (AE) may augment secondary injury processes following TBI and polytrauma (Price et al., 2024, 2025; Proctor et al., 2025; Skovira et al., 2016; Wallen et al., 2022). A recent porcine study by our group on TBI with 40% HS (TBI + HS) reported modest increases in cerebral perfusion pressure and inflammation after real (non-simulated) tactical AE (low flight profile with high g forces) compared with standard and Mock AE strategies (Andrew R Mayer et al., 2026). Given the diverse roles of the KP, including inflammatory mechanisms and energy metabolism, the current follow-up study examined the KP in the same animal cohort supplemented with additional sham animals from a separate study (Andrew R. Mayer et al., 2026; van der Horn et al., 2026).

The first study objective was to determine the effects of TBI + HS on KP metabolites, focusing on KYNA and QUIN as the main neuroactive metabolites linked to inflammation, and 3-HK as a marker of oxidative stress. We hypothesized rapid and sustained alterations in these metabolites after TBI + HS relative to the sham cohort, consistent with KP activation, persisting up to 24 h (final sampling time point), characterized by increased serum QUIN and KYNA, reduced neuroprotective KYNA/QUIN ratios and elevated 3-HK. We expected KP activation, and elevated QUIN in particular, to be associated with microglial activation in post-mortem brain tissue assessed using ionized calcium-binding adaptor molecule 1 (Iba1) immunohistochemistry. The second study objective was to assess whether different AE strategies modulate KP metabolites (as in (Andrew R Mayer et al., 2026)). We hypothesized that tactical evacuation would result in higher QUIN and 3-HK concentrations and lower KYNA/QUIN ratios than standard evacuation and Mock flight conditions. Additional exploratory analyses examined the relationship of KP metabolites with biomarkers of systemic shock (i.e., lactate, glucose, and bicarbonate), TBI biomarkers (glial fibrillary acidic protein [GFAP], ubiquitin carboxy-terminal hydrolase L1 [UCH-L1] and neurofilament light chain [NfL]).

## 2 Methods

### 2.1 Study cohort and experimental procedures

The study was conducted in accordance with the Animal Research: Reporting In Vivo Experiments (ARRIVE) 2.0 guidelines (Percie du Sert et al., 2020) as previously reported (Andrew R Mayer et al., 2026). All animal procedures were approved by the local Institutional Animal Care and Use Committee (IACUC) and the U.S. Army Medical Research and Materiel Command (USAMRMC) Animal Care and Use Review Office (ACURO) prior to study initiation. Twenty-four sexually mature Yucatan swine (vendor: Premier BioSource) were randomly assigned using blocked randomization based on sex and weight to one of three conditions: tactical evacuation flight (TEF), standard evacuation flight (SEF), or Mock flight between October and November 2023 (Andrew R Mayer et al., 2026). One additional animal was maintained as a spare. Sample size selection was informed by prior publications available at the time of the Department of Defense funding application. Sham animals were obtained as part of another study (Andrew R. Mayer et al., 2026; van der Horn et al., 2026).

A 14-day quarantine period, during which no experimental procedures were performed, allowed the animals to acclimate to the study altitude (∼5400 feet above sea level) before the start of the experimental procedures. Prior to surgical procedures, animals were fasted for 6–12 h while maintaining *ad libitum* access to water. Animals were initially sedated with midazolam (0.5 mg/kg, IM) and premedicated with buprenorphine (0.12 mg/kg, SQ). Following sedation, animals were intubated and maintained under general anesthesia using isoflurane (5.0% for induction and 0.5–5.0% for maintenance in combination with oxygen). A propofol bolus (0.8–1.5 mg/kg IV) was administered as needed during surgical and experimental procedures. For TBI + HS animals, central venous and arterial access was established by attempting catheter placement in both femoral arteries, with venous catheterization achieved in a subset of animals when arterial placements failed. Local anesthetic (lidocaine/bupivacaine; maximum 2 mg/kg) was administered as needed to facilitate catheter placement. The femoral artery was used for continuous monitoring of mean arterial pressure (MAP) and for point-of-care (POCT) blood sampling, while either the femoral artery or vein were used during controlled blood loss procedures. TBI + HS animals also received a NeuroVent PTO intracranial pressure (ICP) probe (Raumedic AG, Helmbrechts, Germany), which was secured using a sterile polymer bolt affixed to the skull with a twist drill. Additional intravenous access was obtained via the auricular vein for anesthesia administration. Sham animals received only one femoral vein catheter for blood sampling and no ICP probe but otherwise underwent similar pre-trauma procedures.

The experimental procedures and timeline are depicted in Fig. 1. The TBI + HS model used in this study was consistent with previously described protocols (Andrew R Mayer et al., 2026). A closed-head rotational TBI was induced using a pneumatic device (HYGE, Inc., Kittanning, PA, USA), targeting a rotational velocity of 250 radians/second in the coronal plane. Animals were secured to the linkage assembly using two straps connected to a bite bar, with additional protective measures applied to reduce the risk of maxillofacial injury (Keating et al., 2020). Isoflurane administration was discontinued approximately 30s prior to TBI induction. Head kinematics were recorded using a combined triaxial linear accelerometer and triaxial angular rate sensor, while an identical separate sensor recorded device kinematics. Approximately 2–3 min following TBI, animals underwent controlled arterial hemorrhage targeting 40% of the estimated total blood volume (eTBV), calculated as body weight × 65 mL/kg. Blood loss was performed in a biphasic manner, and 20% of the collected blood (i.e., 50% of what was extracted) was stored in an anticoagulant (CPDA-1) bag for later autologous transfusion. Data on head kinematics was reported in our previous publication and were equivalent across sub-groups (Andrew R Mayer et al., 2026).

**Fig. 1:**
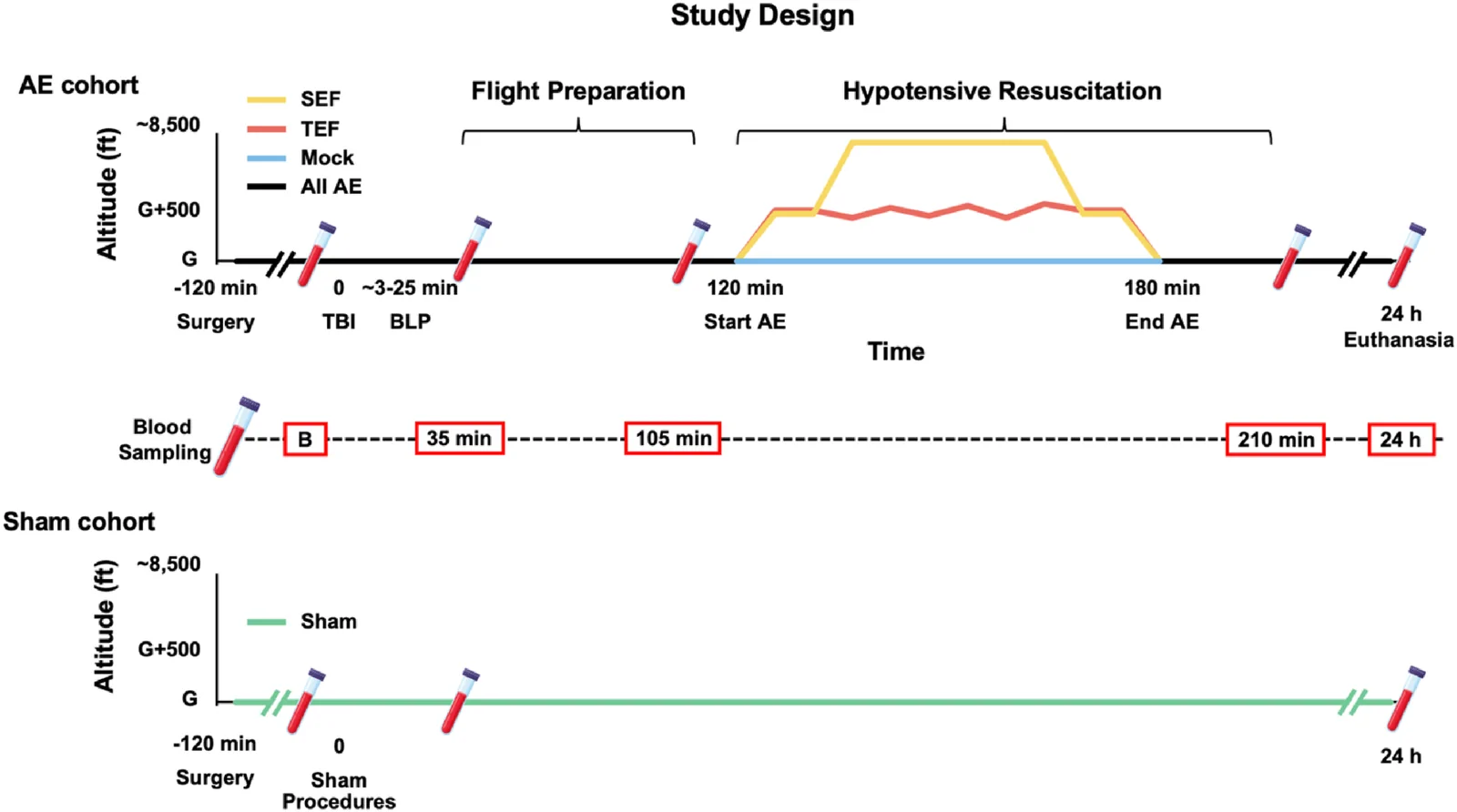
Infographic depicting experimental procedures and timeline. The study included two cohorts: an aeromedical evacuation (AE) cohort and a sham cohort derived from a separate study (Andrew R. Mayer et al., 2026; van der Horn et al., 2026). In the AE cohort, conditions included a standard evacuation flight (SEF; yellow trace), a tactical evacuation flight (TEF; red trace), and a Mock condition (blue trace; stationary at ground level), with shared procedures indicated by the black line. The flight profile is presented as altitude (feet above sea level), including study site ground level (G; ∼5400 ft). Blood samples (depicted as blood tubes on the timeline) were obtained at baseline (B), 35 min after traumatic brain injury (TBI), 105 min post-TBI (immediately before flight), 210 min post-TBI, and 24 h post-TBI. The blood loss phase (BLP) occurred immediately following TBI, lasting for approximately 23 min. The sham cohort (green trace) underwent sham procedures without TBI induction and without blood loss, with blood sampling performed at B, 35 min, and 24 h post-procedure. *Abbreviations:* AE = aeromedical evacuation; BLP = blood loss phase; HS = hemorrhagic shock; SEF = standard evacuation flight; TBI = traumatic brain injury; TEF = tactical evacuation flight.

After completion of blood loss, anesthesia was transitioned from isoflurane to midazolam (continuous-rate infusion, 0.5–1.5 mg/kg/h) and/or ketamine (8–33 mg/kg/h) to minimize depressive effects on cardiovascular and respiratory function. During this phase, supportive care was intentionally limited to warming blankets to simulate an austere care environment. Animals were subsequently instrumented with additional sensors and monitored in preparation for AE procedures, which began 120 min after TBI. At that time, warmed autologous shed blood products were rapidly reinfused, and supportive measures including warming blankets and assisted ventilation with supplemental oxygen were initiated in accordance with the 2023 U.S. Army Aeromedical Evacuation Standard Medical Operating Guidelines (SMOG) for critical care flight paramedics.

All flights were conducted using a U.S. Army HH-60M Black Hawk medical evacuation helicopter. The SEF profile involved an immediate climb followed by straight and level flight at approximately 8500 feet and ∼100 knots, and then descent, with a total duration of 60 min. The TEF profile consisted of flight at a maximum altitude of approximately 500 feet above ground level and included tactical maneuvers such as maximum velocity flight, cyclic climbs, and sharp banking before descent. Animals assigned to the Mock transport condition remained stationary in the flight bay for the same 60-min period.

Serum and plasma samples were collected for the TBI + HS cohort at baseline, 35 min, 105 min (pre-flight), 210 min (post-flight), and 24h post-TBI (terminal time point). Additional blood-based physiological biomarkers were measured using a POCT device (i-STAT, CG8+ cartridges). From this panel, lactate, glucose, and bicarbonate (HCO_3_^−^) were selected as primary markers for further analyses related to the KP, similar to our previous publication (Andrew R Mayer et al., 2026), which also reports these physiological data. For one Mock animal, there were no 24 h serum samples available.

For sham animals, serum and plasma samples were collected at baseline, 5 min, 35 min, 150 min, 24 h, and 7 days following sham procedures (Andrew R. Mayer et al., 2026; van der Horn et al., 2026). Only the baseline, 35 min, and 24 h time points were included in the present study, as these correspond to shared time points with the TBI + HS cohort.

All surviving animals in the TBI + HS model were sedated and then euthanized with an injection of a pentobarbital-based euthanasia solution (2.0mL/4.5 kg, IV) at 24 h post-injury; sham animals were euthanized using the same procedure at 7 days post-surgical procedures.

### 2.2 Blood analyses

Blood samples for biomarker analysis were centrifuged for 10 min at 2500 relative centrifugal force, aliquoted into 400–500 μL tubes, and immediately stored at −80 °C. Kynurenine analyses were performed at the University Medical Center Groningen (UMCG), the Netherlands (also see (van der Horn et al., 2026)). Analyses of TBI proteins were conducted at the Boston Children's Hospital, Boston, MA, U.S.A. Samples were shipped from Albuquerque to Boston or Groningen on dry ice, with confirmation that samples remained frozen upon arrival. Laboratory personnel were blinded to group allocation.

Liquid chromatography coupled with isotope dilution tandem mass spectrometry (LC–MS/MS) was used to quantify KYNA, QUIN, and 3-HK, as well as TRP and KYN, as previously described (Ford et al., 2007; Van Faassen et al., 2020); because TRP and KYN were not a primary focus of the study, their results are reported in the Supplementary Materials. The assay was validated according to our laboratory method validation procedure based on the Dutch guidelines for validation of analytical methods in medical laboratories by the Dutch Society of Clinical Chemistry and Laboratory Medicine (NVKC werkgroep Verificatie & Validatie, 2017). In short, the following parameters were validated using quality control samples at three different concentrations where appropriate: imprecision (intra- and inter-assay imprecision, n = 10), recovery, linearity, carry-over (CLSI, 2025), and limit of quantification. Unlabeled standards of each metabolite were purchased from Merck (Darmstadt, Germany). Stock solutions were prepared and diluted until a final calibration curve was constructed from a working solution, which was made fresh on the day of analysis with concentration ranges from: 15 – 602 nmol/L, 170 – 6804 nmol/L, 13 – 510 nmol, 9.75 – 390 μmol/L and 0.549 – 22.0 μmol/L, for KYNA, QUIN, 3-HK, TRP, and KYN respectively.

For analyses, 50 μL of serum was combined with deuterated (KYNA[^2^H_5_], QUIN[^2^H_3_] or ^13^C-labeled internal standards (3-HK[^13^C_6_], TRP[^13^C_11_], KYN[^13^C_6_]), followed by derivatization and incubation for 30 min. The reaction was terminated with trichloroacetic acid, after which proteins were precipitated. Following centrifugation, 5 μL of the supernatant was injected into an Acquity 2D UPLC system (Waters, Milford, MA, USA) coupled to a Xevo TQ-XS mass spectrometer (Waters, Milford, MA, USA). Total runtime was 10 min. Interassay imprecision was assessed at three concentration levels over 10 different days and was <3.9%, <3.2%, <3.5%, <3.1% and <3.0% for KYNA, QUIN, 3-HK, TRP and KYN, respectively. Limit of quantification was 1.7 nmol/L, 14 nmol/L, 1.6 nmol/L, 0.5 μmol/L and 0.05 μmol/L, for KYNA, QUIN, 3-HK, TRP, and KYN respectively. Recovery was 100% ± 15% for each metabolite. There was no carry-over, and no significant ionization suppression was observed.

TBI protein concentrations in plasma samples were measured in duplicate using a Simoa HD-1 Analyzer (Quanterix, Billerica, MA, USA) with a 4-plex assay for NfL, GFAP, and UCH-L1 (See (Andrew R Mayer et al., 2026)). Tau demonstrates limited cross-species reactivity for this assay (Mayer et al., 2021).

### 2.3 Iba1 immunohistochemistry

Brain histopathology was performed on 8-μm thick sections at the level of the head of the posterior hippocampus. Single immunohistochemistry (see Supplemental Materials) labeling was performed for microglia using Iba1 (WAKO FujiFilm, Osaka, Japan; 1:15k). Whole slide images were obtained using a Zeiss Axioscan 7 slide scanner and manually annotated to delineate white and gray matter, as well as to exclude tissue artifacts. Quantification of microglia was performed for total white matter within each slice. Specifically, stain intensity thresholds were optimized for Iba1 through visual inspection by an experienced rater using multiple animals and positive and negative controls from antibody testing. Threshold settings were defined such that the “weak” intensity threshold of Iba1 staining primarily captured microglial processes, while the “stronger” intensity threshold primarily identified microglia soma. Details on these analyses can be found in the Supplementary Materials. For one Mock AE animal, there was no Iba1 data available.

### 2.4 Statistical plan

All analyses were conducted using IBM SPSS Statistics v30. Animal (baseline) characteristics were compared between groups (TEF, SEF, Mock AE and sham) using univariate analysis of variance (age, weight) or Chi square tests (sex).

All KP metabolites were natural log-transformed before statistical analyses. Distributions were inspected after transformation to verify approximate normality. There were no extreme outliers (3×IQR above the 3rd or below the 1st quartile) post-transformation. Therefore, no data were removed.

Regarding the first objective of examining the effects of TBI + HS on KP metabolites, the following two sets of linear mixed-effects (LME) models were performed: (a) primary models including Time (baseline, 35 min, 105 min, 210 min, 24h) to examine KP time courses post-injury in the TBI + HS group (with 1st order autoregressive [AR1] covariance structure) and (b) secondary 2 × 2 (Group [TBI + HS vs. Sham] × Time [35 min, 24 h]) models including baseline concentration, age and weight as covariates (with compound symmetry [CS] covariance structure), to examine whether polytrauma increases KP metabolites relative to sham for shared time points. Significant main effects of Time or Group × Time interactions were followed up with pairwise comparisons or general linear models (GLM) as appropriate. Partial eta squared (η_p_^2^) effect sizes were computed for follow-up GLMs.

The potential moderating effects of AE strategy on KP metabolites post-flight were examined with 3 × 2 (AE strategy [TEF vs. SEF vs. Mock AE]) × Time [210 min vs. 24 h]) LME models, including pre-flight (105 min) concentration as a covariate (similar to (Andrew R Mayer et al., 2026)) and CS covariance structure. Significant AE strategy × Time interactions were followed up with GLMs as appropriate.

Percentages of positive area for Iba1 staining at “weak” (microglial processes) and “stronger” (microglial soma) intensity thresholds were natural log transformed and compared between TEF, SEF and Mock groups using GLMs. To examine for potential neuroprotective or deleterious effects on subsequent neuroinflammation, partial correlations between KP metabolites (KYNA, QUIN, KYNA/QUIN ratio, and 3-HK) and Iba1 staining were computed at each time point, adjusting for baseline metabolite levels.

Additional exploratory LME models were conducted to examine associations of KP metabolites (dependent variable) with (1) physiological biomarkers of systemic shock (lactate, glucose, and bicarbonate), and (2) biomarkers of TBI (GFAP, UCH-L1, and NfL) in the TBI + HS cohort. These independent variables were z-scored prior to entry into the model. Time (baseline, 35 min, 105 min, 210 min, 24h) was included as additional factor and AR1 covariance structure was used.

Considering the preliminary nature of the study and the small sample size for AE groups, statistical results were considered significant at *P* < 0.05 and were not corrected for multiple comparisons (similar to (Andrew R Mayer et al., 2026)). Therefore, all results should be interpreted as exploratory and require confirmation in independent datasets.

## 3 Results

### 3.1 Animal and injury characteristics

Three swine (one male) from the TEF group, one (female) swine from the SEF group, and one (male) swine from the Mock group either did not survive the trauma procedures or were excluded because of procedural deviations prior to AE. All five animals expired as a result of respiratory arrest. Consequently, the spare animal and one animal initially assigned to the Mock transport condition were reallocated to the TEF group. The final TBI + HS sample (N = 20) therefore consisted of seven TEF, seven SEF and six Mock AE animals. There were no significant differences in age, weight and sex between these AE sub-groups (*P*'s > 0.05). However, age and weight were significantly lower in the total TBI + HS sample as compared to sham animals (Table 1). Sex was not significantly different between groups.

**Table 1:** Animal characteristic data.

	Polytrauma (TBI + HS) (N = 20)	Sham (N = 10)	*p*-value
Age, days	198.9 (5.57)	221.7 (35.5)	0.008
Sex, n (% female)	9 (45)	5 (50)	0.796
Weight, kg	24.0 (3.0)	28.9 (3.3)	<0.001

*Notes*: Data for age and weight are formatted as mean ± standard deviation.

*Abbreviations*: HS = hemorrhagic shock; TBI = traumatic brain injury.

### 3.2 Effects of TBI + HS on the KP

For the TBI + HS cohort, significant main effects of Time were observed for KYNA (*F*_4,71.95_ = 50.78, *P* < 0.001), QUIN (*F*_4,71.85_ = 10.48, *P* < 0.001), KYNA/QUIN ratio (*F*_4,69.82_ = 36.44, *P* < 0.001) and 3-HK (*F*_4,65.87_ = 11.24, *P* < 0.001; Fig. 2). Follow-up pairwise comparisons showed that KYNA (*P*'s < 0.003) and the KYNA/QUIN ratio (*P*'s < 0.014) increased significantly at 35 min, 105 min, and 210 min as compared to baseline. For QUIN, concentrations increased significantly at 35 min and 105 min relative to baseline (*P*'s < 0.008). KYNA concentrations and the KYNA/QUIN ratio were significantly lower at 210 min compared to 35 min and 105 min (all *P*'s < 0.001). For 3-HK, concentrations at 210 min were higher than at 35 min (*P* = 0.011), and 105 min (*P* = 0.002). At 24 h, 3-HK, KYNA, and KYNA/QUIN ratio were significantly reduced compared with earlier time points (*P*'s < 0.025), while QUIN did not differ from baseline (*P* = 0.307). Results for KYN/TRP ratio, a potential surrogate marker of IDO1 and TDO2 activity, are reported in the Supplementary Materials. Summary metrics for all metabolites and ratios are listed by group and time point in Table S1.

**Fig. 2:**
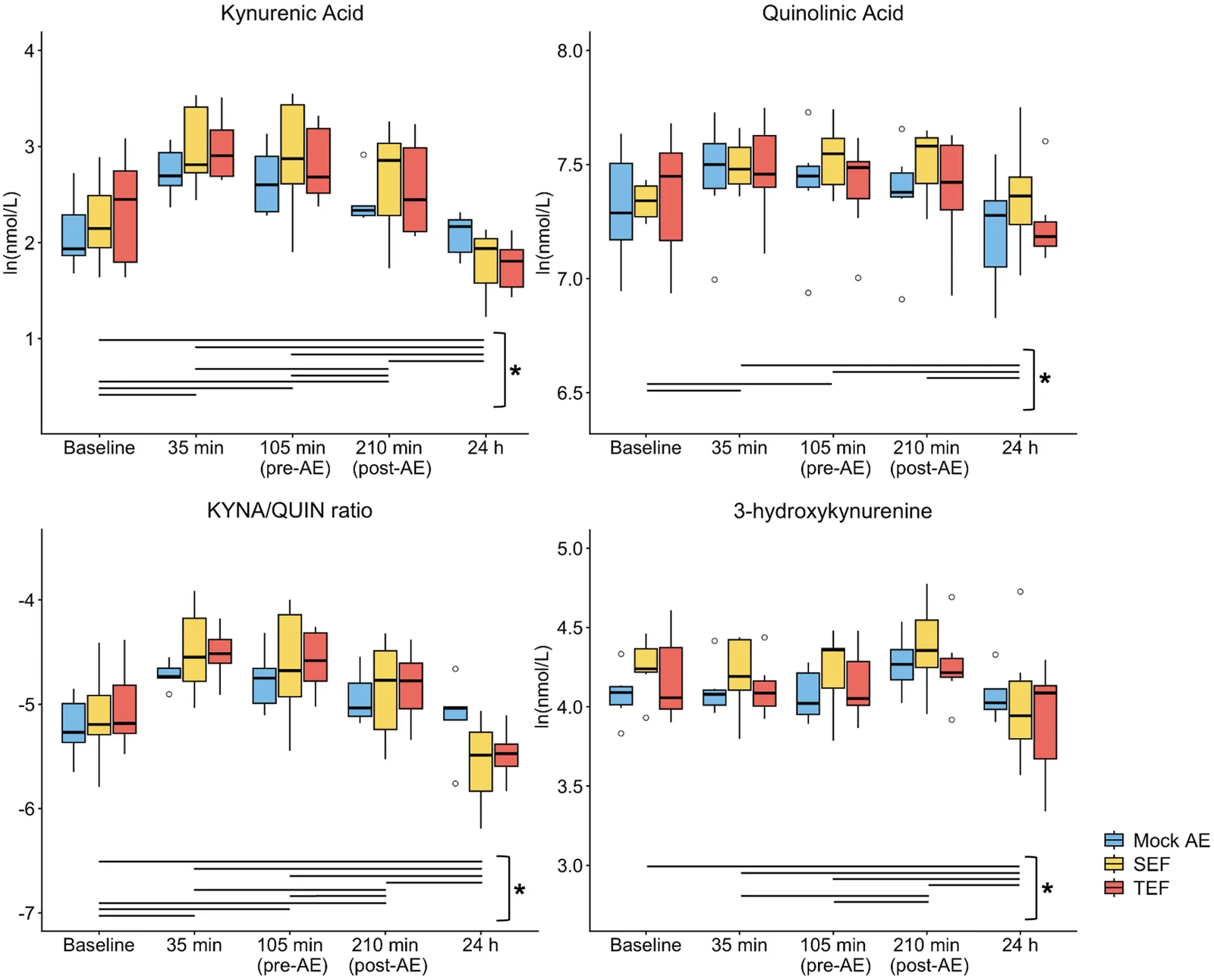
**Kynurenine pathway metabolites over time in animals with polytrauma and aeromedical evacuation.** Summary measures are shown separately for each aeromedical evacuation (AE) group. For each metabolite and ratio, a significant main effect of Time was observed in the traumatic brain injury and hemorrhagic shock (TBI + HS) cohort. Lines adjacent to the asterisks show significant follow-up pairwise differences between time points for the entire TBI + HS cohort. Outliers (1.5*interquartile range [IQR] above 3rd quartile/below 1st quartile) are indicated with open circles (there were no extreme outliers, defined as 3*IQR above 3rd/below 1st quartile). *Abbreviations:* AE = aeromedical evacuation; HS = hemorrhagic shock; KYNA = kynurenic acid; QUIN = quinolinic acid; SEF = standard evacuation flight; TBI = traumatic brain injury; TEF = tactical evacuation flight.

Models comparing TBI + HS to sham animals showed a significant Group × Time interaction for KYNA (*F*_1,27.8_ = 9.5, *P* = 0.005), QUIN (*F*_1,27.9_ = 4.98, *P* = 0.034), and KYNA/QUIN ratio (*F*_1,27.7_ = 6.83, *P* = 0.014), after adjusting for age, weight and baseline concentration. Follow-up tests indicated significantly increased KYNA (*P* < 0.001, η_p_^2^ = 0.36), QUIN (*P* < 0.001, η_p_^2^ = 0.58), and KYNA/QUIN (*P* = 0.011, η_p_^2^ = 0.23) in TBI + HS vs. sham animals at 35 min (Fig. 3) but not at 24h post-injury, confirming results for the within-subject models. Given the large baseline differences, a significant main effect of group for 3-HK (*F*_1,25.92_ = 33.04, *P* < 0.001) was observed only when baseline concentration was not included as a covariate in the model. Results for KYN/TRP ratio are reported in the Supplementary Materials. Summary metrics for all metabolites and ratios are listed in Table S1.

**Fig. 3:**
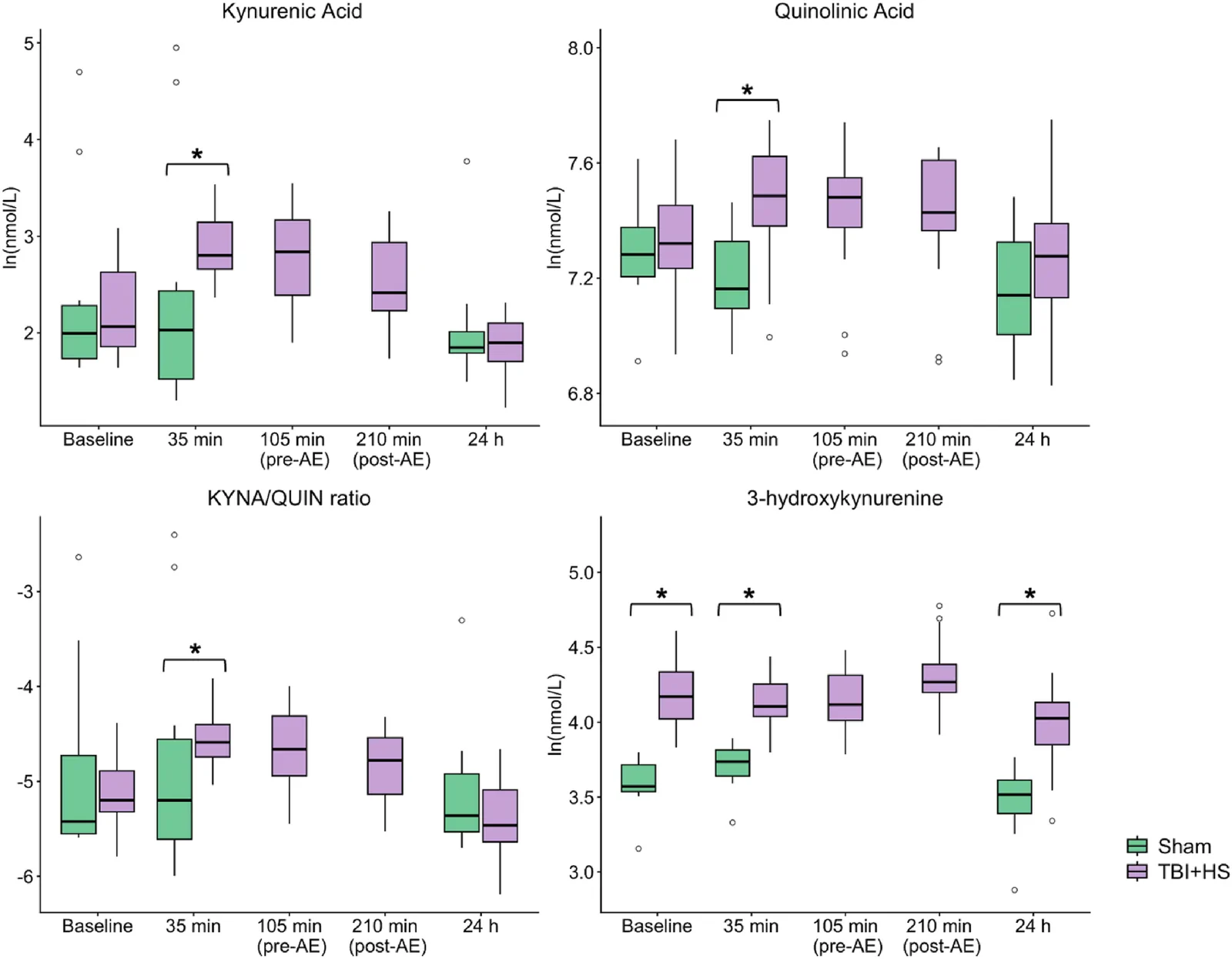
**Kynurenine pathway metabolites in polytrauma relative to sham animals.** Asterisks depict significant differences between traumatic brain injury and hemorrhagic shock (TBI + HS) and sham cohorts, while correcting for group differences in baseline concentrations, age and weight. For 3-hydroxykynurenine, large group differences were found at baseline, persisting up to 24 h; however, these differences were only significant when baseline concentration was not included as a covariate in the model. Outliers (1.5*interquartile range above 3rd quartile/below 1st quartile) are indicated with open circles (there were no extreme outliers, defined as 3*IQR above 3rd/below 1st quartile). *Abbreviations:* AE = aeromedical evacuation; HS = hemorrhagic shock; KYNA = kynurenic acid; QUIN = quinolinic acid; SEF = standard evacuation flight; TBI = traumatic brain injury; TEF = tactical evacuation flight.

### 3.3 Effects of aeromedical evacuation on the KP

A significant AE strategy × Time interaction was observed for KYNA (*F*_2,16.4_ = 4.13, *P* = 0.035) post-flight after adjusting for preflight (105 min) concentration. Follow-up tests revealed a significant effect at 24h (*P* = 0.046, η_p_^2^ = 0.34) but not at 210 min (*P* > 0.05), with lower concentrations of KYNA in both TEF and SEF as compared to Mock AE (*P*'s < 0.05). Results for 3-HK, QUIN, and KYNA/QUIN ratio were not significant. Results for KYN/TRP ratio are reported in the Supplementary Materials. Summary metrics for all metabolites and ratios are listed in Table S1.

### 3.4 Iba1 results

GLMs indicated that there were no significant differences (*P*'s > 0.05; Fig. 4, panel b) in percentages of Iba1 positive brain tissue area between the Mock AE, SEF and TEF groups for either microglial processes (classified by “weak” intensity staining; yellow areas in thresholded images in panels d-f in Fig. 4) or soma (classified by “stronger” intensity staining; orange areas in thresholded images). Additional models examining the relationship between KP metabolites and immunohistochemical measures of inflammation at 24 h post-injury showed that KYNA and the KYNA/QUIN ratio were uniquely (i.e., partial correlations controlling for baseline concentrations) and negatively associated with microglial processes and soma staining when KP concentrations were measured at 35, 105, and 210 min post-injury. These associations were no longer significant when KP concentrations at 24 h were considered. Panel c in Fig. 4 displays the partial correlation coefficients (left y-axes, solid lines) across KP sampling time point, all in relation to Iba1 measured at 24 h. For plotting purposes, *P*-values for correlations were log_10_-transformed and multiplied by −1, such that higher values correspond to lower *P*-values; these were plotted using dashed lines and on the right y-axes. No significant associations were found for QUIN and 3-HK.

**Fig. 4:**
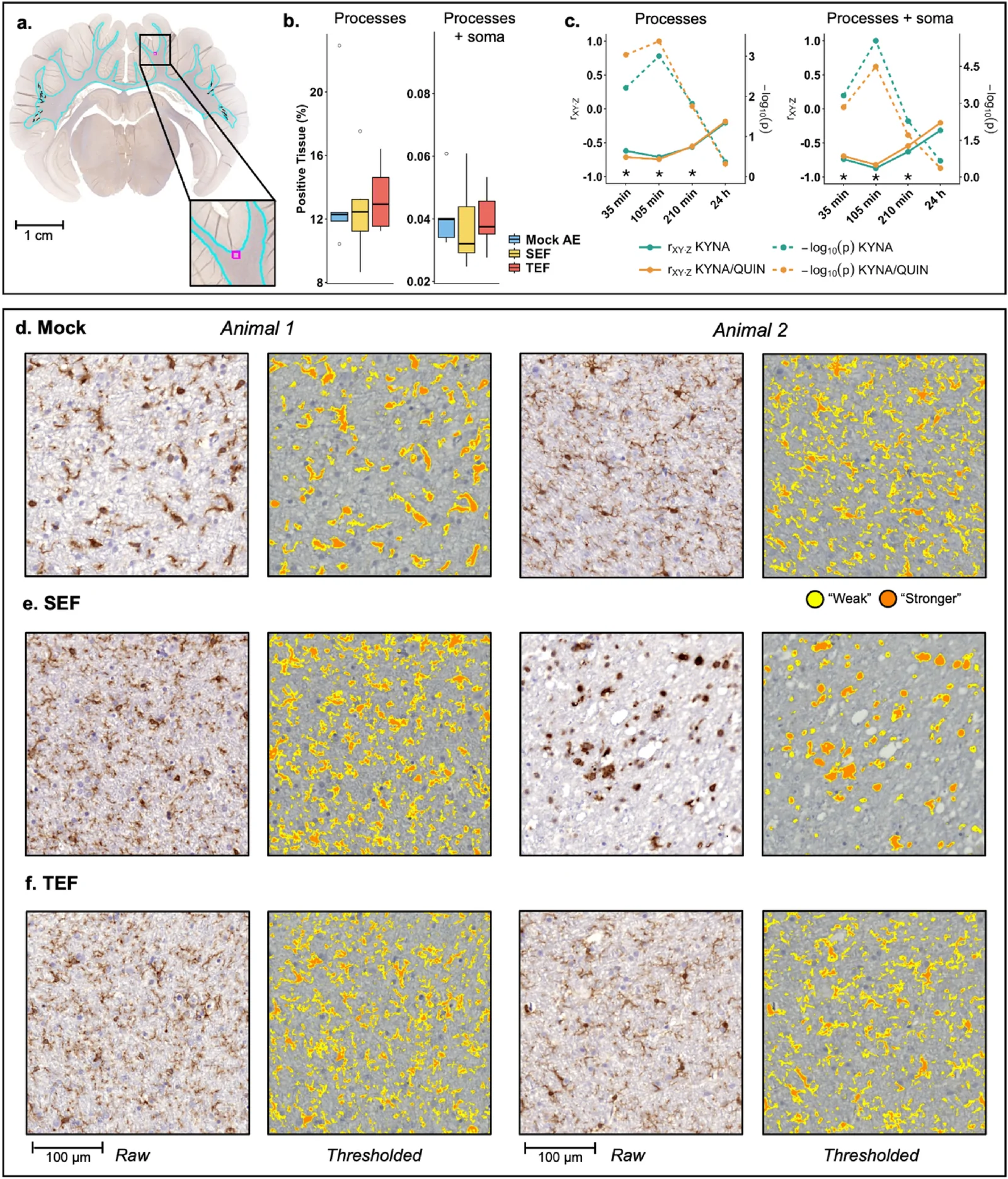
**Immunohistochemistry of microglia reactivity.** a) White matter (cyan trace) at the level of the hippocampus, including region of interest from which individual animal data are presented (panels d-e). Regions with artifacts were also manually traced (see black tracing within white matter) and excluded from all subsequent analyses. b) Boxplots showing the percent positive area of ionized calcium binding adaptor molecule 1 (Iba1) staining for the Mock, standard evacuation flight (SEF) and tactical evacuation flight (TEF) groups. c) Partial correlations (r_XY.Z_; left y-axes, solid curves) of KYNA and KYNA/QUIN with microglial staining, while correcting for baseline KYNA and KYNA/QUIN values, as well as the corresponding -log_10_ transformed p-values (right y-axes, dashed curves, higher p-values indicating higher significance). Asterisks show significant correlations (*P* < 0.05). d-e) Raw and thresholded Iba1 images for two animals from the Mock, SEF and TEF groups, where “weak” staining primarily corresponded to microglial processes and “stronger” staining primarily corresponded to cell soma. *Abbreviations*: Iba1 = ionized calcium binding adaptor molecule 1; KYNA = kynurenic acid; QUIN = quinolinic acid; SEF = standard evacuation flight; TEF = tactical evacuation flight. (For interpretation of the references to colour in this figure legend, the reader is referred to the Web version of this article.)

### 3.5 Associations with biomarkers of systemic shock and TBI

In the TBI + HS cohort, KYNA and the KYNA/QUIN ratio showed distinct associations with biomarkers of systemic shock and TBI, including positive relationships with lactate, GFAP, and UCH-L1, and inverse associations with NfL (Table 2). For 3-HK, inverse associations were observed with lactate, bicarbonate, and NfL.

**Table 2:** Linear mixed model results for the relationships of kynurenine pathway activation with biomarkers of shock and TBI in swine with TBI + HS.

	KYNA	QUIN	KYNA/QUIN	3-HK
Lactate	*P* = 0.014 *β* = 0.2	N.S.	*P* = 0.025 *β* = 0.17	*P* = 0.006 *β* = −0.16
Glucose	N.S.	N.S.	N.S.	N.S.
Bicarbonate	N.S.	N.S.	N.S.	*P* = 0.012 *β* = −0.15
GFAP	N.S.	N.S.	*P* = 0.035 *β* = 0.08	N.S.
UCH-L1	*P* = 0.015 *β* = 0.14	N.S.	*P* = 0.039 *β* = 0.11	N.S.
NfL	*P* = 0.027 *β* = −0.14	N.S.	N.S.	*P <* 0.001 *β* = −0.15

*Notes*: Data show *P*-values and estimates reflecting magnitude of change in kynurenine pathway metabolite (across the entire time course) in response to one standard deviation increase in biomarkers.

*Abbreviations*: 3-HK = 3-hydroxykynurenine; GFAP = glial fibrillary acidic protein; KYNA = kynurenic acid; NfL = neurofilament light; N.S. = not significant; QUIN = quinolinic acid; UCH-L1 = ubiquitin carboxy-terminal hydrolase L1.

## 4 Discussion

The current study demonstrates robust findings (i.e., large effect sizes) that are consistent with early KP activation in a TBI + HS polytrauma model, as hypothesized. Contrary to our hypotheses, the KP was not persistently activated at 24 h, and the KYNA/QUIN ratio was increased instead of decreased, which may reflect enhanced utilization of QUIN under conditions of metabolic stress. Furthermore, the effects of AE were modest and confined to decreased KYNA (instead of increased QUIN) for both evacuation groups suggesting that the benefits of evacuation flights likely outweigh any potential adverse effects. Strong negative correlations were found between KYNA concentrations and microglia activation, as assessed by Iba1 staining of post-mortem brain tissue, which is consistent with a potential immunomodulatory role of KYNA following TBI + HS. Associations between KP metabolites with biomarkers of both systemic shock and TBI were observed, which may reflect processes related to metabolic stress and blood-brain barrier (BBB) breach.

Relative to baseline, concentrations of KYNA and QUIN, as well as the KYNA/QUIN ratio, were significantly increased at 35 min following TBI + HS, remained elevated up to 210 min, and returned to or fell below baseline levels by 24 h. The early elevations at 35 min were confirmed by comparisons with sham animals. These results are consistent with rapid KP activation that can be explained by the pronounced stress (Rizoli et al., 2017; Watts and Westfall, 1964; Woolf et al., 1987; Zhao et al., 2024) and inflammatory response (Li et al., 2026) to trauma, which increases available free TRP (∼90% of circulating TRP is albumin-bound (Badawy and Guillemin, 2022)). Catecholamines and inflammatory stimuli promote lipolysis, leading to elevated levels of non-esterified fatty acids that displace TRP from albumin, thereby increasing circulating free TRP concentrations and its availability for metabolism through the KP (Badawy and Evans, 1976; Foley et al., 2021; Höglund et al., 2019; Rittig et al., 2016). Although increased KYNA and QUIN clearly suggest activation of the KP, this was not quantitatively supported by an increase in the KYN/TRP ratio (see Supplementary Materials). However, this ratio may not be a reliable proxy for IDO1 and TDO2 activity, as it is influenced by multiple factors, including the flux of plasma free TRP down the KP and activity of downstream KP enzymes (preventing KYN accumulation) (Badawy and Guillemin, 2019). Furthermore, increased availability of free TRP would not necessarily change serum TRP concentrations, as total TRP (free and albumin-bound) was measured in our study.

Contrary to our hypothesis, KYNA/QUIN ratios were increased instead of decreased. A proportionally greater rise in KYNA than QUIN may reflect a shift toward a neuroprotective rather than neurotoxic profile as a potential compensatory response to counteract excitotoxicity driven by elevated QUIN and glutamate release after TBI (Meier and Savitz, 2022; Miranda et al., 1997) as well as for systemic inflammatory effects (Steiner et al., 2014; Wirthgen et al., 2018). However, we deem it more likely that increased KYNA/QUIN ratios reflect a relative reduction in QUIN compared to KYNA as available QUIN is increasingly used for NAD^+^ synthesis required for ATP production via glycolysis. In fact, the KP preferentially follows the arm leading to QUIN production, and thus NAD^+^ synthesis could be considered the most important function of the KP (Braidy et al., 2011; Liu et al., 2007). Under hypoxic conditions, such as hemorrhagic shock, impaired mitochondrial electron transport chain activity (as part of oxidative phosphorylation) can limit NAD^+^ regeneration, making NAD^+^ availability rate-limiting for sustaining glycolysis (Sims et al., 2018). Thus, QUIN after TBI + HS may, in part, be diverted to support NAD^+^ homeostasis during metabolic stress, potentially contributing to the observed increase in the KYNA/QUIN ratio. Additionally, enzymes in the route toward QUIN may become saturated at high KYN concentrations, resulting in a relative diversion of KYN toward the kynurenine aminotransferases (KAT) enzymes which produce KYNA (Badawy, 2017; Pocivavsek et al., 2024; Schwarcz et al., 2012). These KAT enzymes exhibit relatively low substrate (i.e., KYN) affinity, reflected by high Michaelis constants (*K*_m_), meaning that higher substrate concentrations are required for the reaction rate to reach half of its maximum.

Links to metabolic stress in polytrauma are further supported by the positive associations of KYNA and the KYNA/QUIN ratio with lactate observed in the current TBI + HS study. Both TBI and HS are characterized by stress-induced hyperglycemia that fuels increased glycolytic flux, leading to lactate accumulation, all of which are associated with injury severity and mortality (Åkerlund et al., 2022; Broder and Weil, 1964; Carpenter et al., 2015; Deininger et al., 2026; Eakins, 2009; Gribnau et al., 2024; Kreutziger et al., 2015; Martin-Rodriguez et al., 2024; Oft et al., 2024). Consistent with this, our previous work in the same cohort demonstrated that glucose and lactate levels peak at 35 min post-injury, accompanied by low bicarbonate, with subsequent acidosis followed by compensation (partly through resuscitation) (Andrew R Mayer et al., 2026). During hypoperfusion, conversion of the glycolytic end product pyruvate to lactate, catalyzed by lactate dehydrogenase, regenerates NAD^+^ from NADH, thereby sustaining glycolysis, which becomes the primary ATP source when oxygen is limited.

Similarly, following TBI, the brain also shifts to a state of relative hyper-glycolysis as mitochondrial oxidative metabolism is impaired (Oft et al., 2024), a process that may be further exacerbated by hypotension (Åkerlund et al., 2022; Lee et al., 2024; McHugh et al., 2007). Experimental data indicate that lactate, via conversion back to pyruvate by lactate dehydrogenase, can serve as a co-substrate to promote KYNA production in the brain during states of metabolic stress, when the primary co-substrate for KAT enzymes, α-ketoglutarate derived from the (aerobic) tricarboxylic acid cycle, is limited (Hodgkins PS and Schwarcz R, 1998). This is consistent with the relationship between lactate and KYNA observed in the current study. Interestingly, 3-HK increased only modestly at 210 min relative to earlier time points, suggesting limited accumulation of this redox-active intermediate. Elevated baseline 3-HK levels are likely driven by the initial inflammatory response induced by surgical procedures (including ICP probe placement). Future studies should consider collecting blood samples before surgical procedures and assessing additional markers of redox balance (collectively referred to as the “reactive species interactome”) to further elucidate the relationship between 3-HK, oxidative stress and antioxidant defense mechanisms (Visser et al., 2026).

Both standard and tactical evacuation flights were associated with lower KYNA concentrations at 24 h post-injury as compared to Mock flight. The mechanisms underlying this decrease remain speculative, particularly given the relatively long unsampled interval between 210 min and 24 h. Beyond its NMDA receptor-antagonist properties, KYNA exerts anti-inflammatory (Steiner et al., 2014; Wirthgen et al., 2018) and anti-oxidative effects (Lugo-Huitrón et al., 2011; Wirthgen et al., 2018); the observed reduction may therefore reflect increased utilization of KYNA in response to pro-inflammatory and oxidative processes triggered by hypobaria (standard evacuation) and mechanical stress (tactical conditions). Animals were studied, after acclimatization, at an initial altitude of approximately 5400 feet above sea level, with further increases of ∼3000 feet during standard or ∼500 feet during tactical evacuation flights. Prior porcine studies of simulated aeromedical evacuation after TBI, have shown that hypobaria alone, in the absence of hypoxia, can enhance pro-inflammatory responses (Price et al., 2024, 2025). In our previous work in the same TBI + HS cohort, only tactical and not standard evacuation elicited a pro-inflammatory response compared to Mock conditions (Andrew R Mayer et al., 2026), whereas in the present study both flight profiles were associated with reduced KYNA levels. Depletion of KAT co-substrates (i.e, α-ketoglutarate, pyridoxal-5′-phosphate [active form of vitamin B6], 2-oxoacid and pyruvate) may also have contributed to decreased KYNA production post-flight (Hodgkins PS and Schwarcz R, 1998). Future studies with larger sample sizes and more frequent sampling throughout the post-flight time-period are needed to better delineate KYNA dynamics.

Interestingly, strong negative correlations were found between KYNA and KYNA/QUIN at 35, 105 and 210-min post-injury with the Iba1 positive staining of microglia processes and soma in post-mortem tissue (24 h post-injury), which is consistent with a potential anti-inflammatory role of KYNA following TBI + HS. While we had expected to find positive correlations between QUIN with Iba1, our findings are in line with a study using rat striatal cell cultures demonstrating that KYNA attenuates QUIN-induced upregulation of Iba1 (Pierozan et al., 2018). However, unlike TRP, KYN and 3-HK, KYNA has limited permeability across the BBB through passive diffusion, although this may be increased following TBI or as a result of the surgical procedures performed as part of the study (Skorobogatov et al., 2021). While compartment-specific contributions cannot be distinguished, it is plausible that the marked increase in serum KYNA following TBI + HS reflects not only peripheral changes but also increased central nervous system production. Although the associations that were observed with TBI biomarkers may further suggest some degree of brain specificity in the observed KP alterations in our animal model, these overlapping temporal profiles more likely reflect shared systemic injury-related processes, and therefore these findings should be interpreted with caution. Both TBI and hemorrhagic shock induce systemic inflammation, which activates the KP (Liu et al., 2015; Meier and Savitz, 2022). Furthermore, in addition to TBI, hemorrhagic shock disrupts the BBB (Hao et al., 2012), facilitating protein efflux (Andrew R. Mayer et al., 2026). Nevertheless, we consider further exploration of the KYNA branch of the KP warranted. The negative associations with NfL likely reflect divergent temporal trajectories rather than direct causal relationships, with NfL continuing to rise over time relative to decreasing GFAP and UCH-L1 levels (Andrew R. Mayer et al., 2026; Shahim et al., 2016). Microdialysis studies may offer more insight into the extent to which KP alterations following TBI + HS reflect brain-specific processes versus systemic responses to injury**.**

This study has several limitations that warrant consideration. First, the study cohort did not include groups with isolated severe TBI or isolated hemorrhagic shock, which limits our ability to disentangle their individual contributions to KP activation. Second, as sham animals were derived from a separate study, they underwent different surgical procedures (e.g., no ICP probe, single femoral catheter), resulting in baseline differences in KP activity, and allowing alignment with the TBI + HS group at only three time points. Third, sample sizes were relatively small for the three AE cohorts, both limiting statistical power to detect group differences and potentially limiting the generalizability of findings. In contrast, power was more robust when data were combined across all three AE groups. Therefore, our conclusions regarding KYNA and lack of findings in terms of differential Iba1 expression as a function of AE should be interpreted with caution and confirmed in studies with larger sample sizes. Lastly, the current study focused on a select set of KP metabolites in the acute phase following injury. Future preclinical and clinical studies should investigate long-term outcomes with a broader metabolite profile and their related biochemical pathways.

In conclusion, activation of the KP following polytrauma likely represents a double-edged sword, modulating inflammatory and excitotoxic mechanisms, buffering metabolic stress during hypoperfusion, and exerting both pro- and anti-oxidative effects. Our findings suggest that the additional biochemical impact of evacuation flights is limited, in contrast to prior (simulation) AE studies on TBI and TBI + HS suggesting the opposite (Price et al., 2024, 2025; Proctor et al., 2025; Skovira et al., 2016; Wallen et al., 2022). Future porcine studies should explore targeted manipulations of key KP enzymes to test how shifting the balance between neuroprotective and neurotoxic metabolites influences outcomes. Ultimately, these experimental findings require validation in human cohorts to establish their translational relevance.

## CRediT authorship contribution statement

**Harm J. van der Horn:** Writing – original draft, Visualization, Investigation, Formal analysis, Conceptualization. **Kristin E. Connors:** Writing – review & editing, Visualization, Methodology, Investigation. **Carissa J. Mehos Milliken:** Writing – review & editing, Visualization, Methodology, Investigation. **Upasana Nathaniel:** Writing – review & editing, Data curation. **Koen Visser:** Writing – review & editing, Investigation, Data curation. **Tracey V. Wick:** Writing – review & editing. **Samuel D. Miller:** Writing – review & editing. **Andrew P. Gigliotti:** Writing – review & editing, Methodology, Investigation. **Timothy B. Meier:** Writing – review & editing. **Sophie Erhardt:** Writing – review & editing. **Rachel Kinsler:** Writing – review & editing, Resources, Project administration, Methodology, Investigation, Funding acquisition, Conceptualization. **Claude van der Ley:** Writing – review & editing, Methodology, Investigation, Formal analysis, Data curation. **Martijn van Faassen:** Writing – review & editing, Methodology, Investigation, Formal analysis, Data curation. **Ido P. Kema:** Writing – review & editing, Methodology. **Joukje van der Naalt:** Writing – review & editing. **Rebekah Mannix:** Writing – review & editing, Investigation, Data curation. **Andrew R. Mayer:** Writing – review & editing, Validation, Supervision, Software, Resources, Project administration, Methodology, Investigation, Funding acquisition, Formal analysis, Data curation, Conceptualization.

## Ethics declaration

This study was conducted in accordance with the ARRIVE (Animal Research: Reporting of In Vivo Experiments) guidelines. This study was approved by the local Institutional Animal Care and Use Committee (IACUC) and the U.S. Army Medical Research and Materiel Command (USAMRMC) Animal Care and Use Review Office (ACURO).

## Declaration of competing interest

The authors declare the following financial interests/personal relationships which may be considered as potential competing interests: Dr. Meier reports in-kind research support from Abbott and has previously received compensation as a member of the Clinical and Scientific Advisory Board for Quadrant Biosciences Inc.

## Data Availability

Data will be made available on request.
