# Supplementary material for: Kynurenine pathway activation following moderate-to-severe traumatic brain injury with hemorrhagic shock and the effects of aeromedical evacuation
Source: Brain Behav Immun Health. 2026 Oct 13;58:101389. doi: 10.1016/j.bbih.2026.101389 (PMC13694781; doi:10.1016/j.bbih.2026.101389)
Supplement: Supplementary file 1 [file mmc1.docx]

Supplementary Materials for:

**Kynurenine Pathway Activation Following Moderate-to-Severe Traumatic Brain Injury with Hemorrhagic Shock and the Effects of Aeromedical Evacuation**

**Methods**

*Immunohistochemistry*
Brain histopathology was performed on 8-micron thick sections at the level of the posterior hippocampus. Endogenous peroxidase activity was blocked using 3% hydrogen peroxide in water for 15 minutes and antigen retrieval performed in Citrate buffer in a microwave pressure cooker. Sections were then blocked in 1% normal goat serum, incubated at 4 degrees overnight in primary antibody, and incubated with goat anti-rabbit secondary (Vector Labs, BA-1000) and ABC solution the following day (Vector Labs, Burlingame, CA). Sections were visualized with 3, 3’-diaminobenzidine (DAB; Vector Labs, Burlingame, CA), counterstained with hematoxylin, dehydrated, and coverslipped. Positive and negative tissue controls and a no-primary negative control were included in initial antibody testing in order to optimize and validate antigen retrieval times and antibody concentrations. Whole slide images from ionized calcium-binding adaptor molecule 1 (Iba1) and H&E immunohistochemistry were obtained using a Zeiss Axioscan 7 slide scanner with a 20x objective, saved as .czi files, and imported to HALO v4.3.6992.9 (Indica Labs, Albuquerque, NM) for region of interest annotation and Iba1 analysis at a resolution of 0.172um/pixel.

White matter boundaries corresponding to the corpus callosum, radiations of the corpus callosum, and corona radiata were traced by an investigator first in H&E images at 0.5x magnification, then registered to the corresponding Iba1 images. Alignment of registered images was visually assessed and corrected as needed using landmarking tools. WM boundaries were then refined in Iba1 images at 1.5-2.0x magnification as needed for inter-slice variability observed at clearly identifiable morphological boundaries. Areas of overt tissue-processing/post-mortem damage (i.e. tissue tears, folds, or flaking) were excluded by manual annotation.

Area quantification of Iba1 was performed using the Indica Labs Area Quantification v3.0.4 algorithm. To ensure accurate discrimination of positive immunoreactivity while minimizing the inclusion of nonspecific or background staining, threshold stain intensity settings were optimized through visual inspection by two experienced investigators using 7 regions selected across multiple animals within the cohort, blinded to group assignment. Regions were selected as being together representative of the range of staining observed within the cohort. Threshold settings were defined such that the “weak” intensity threshold of Iba1 staining primarily captured microglial processes, while “stronger” intensity thresholds primarily identified microglia soma. Settings were subsequently validated through assessment by a second experienced investigator, such that area and intensity of microglia morphology identified algorithmically were agreed to be consistent with visually identified microglia morphology. Once validated, these threshold settings were applied to all WM annotation layers uniformly across images to maintain consistency and enable reliable quantitative comparisons between samples while minimizing bias, while also excluding any white matter regions demonstrating artifacts via manual tracing (see black tracings on Figure 4a).

**Results**

*Analyses of the kynurenine-to-tryptophan ratio*
There were no significant (*P*’s >0.05) changes in the (natural log-transformed) ratio of kynurenine-to-tryptophan (KYN/TRP) over time within the polytrauma group (**Figure S1**) nor were there significant differences between the polytrauma and sham groups (**Figure S2**), even when baseline concentration was not included as covariate. Furthermore, there were no significant differences (*P*’s >0.05) between aeromedical evacuation groups post-flight.

**Figure S1. Kynurenine/tryptophan ratio over time in animals with polytrauma and aeromedical evacuation.**
Summary measures are shown separately for each aeromedical evacuation (AE) group. Outliers (1.5*interquartile range [IQR] above 3^rd^ quartile/below 1^st^ quartile) are indicated with open circles (there were no extreme outliers, defined as 3*IQR above 3^rd^/below 1^st^ quartile).
*Abbreviations:* AE = aeromedical evacuation; KYN = kynurenine; SEF = standard evacuation flight; TBI = traumatic brain injury; TEF = tactical evacuation flight; TRP = tryptophan.

**Figure S2. Kynurenine/tryptophan ratio over time in polytrauma relative to sham animals.**
Outliers (1.5*interquartile range [IQR] above 3^rd^ quartile/below 1^st^ quartile) are indicated with open circles (there were no extreme outliers, defined as 3*IQR above 3^rd^/below 1^st^ quartile).
*Abbreviations:* AE = aeromedical evacuation; HS = hemorrhagic shock; KYN = kynurenine; TBI = traumatic brain injury; TRP = tryptophan.

**Table S1.** Concentrations and ratios of kynurenine pathway metabolites by time point and group.

|  | **KYNA** | **QUIN** | **KYNA/ QUIN** | **3-HK** | **KYN/ TRP** | **TRP** | **KYN** |
| --- | --- | --- | --- | --- | --- | --- | --- |
| **Baseline** |  |  |  |  |  |  |  |
| *TBI+HS* | 10.18 (1.11) | 1567.52 (72.72) | 0.64  (0.06) | 66.67 (3.25) | 1.33 (0.08) | 46.39 (1.31) | 0.61 (0.03) |
| *Mock* | 8.63 (1.53) | 1539.17 (162.96) | 0.56  (0.07) | 59.76 (4.06) | 1.15 (0.06) | 45.33 (1.72) | 0.52 (0.04) |
| *SEF* | 10.00 (1.69) | 1541.85 (47.37) | 0.65  (0.11) | 71.47 (4.36) | 1.34 (0.12) | 47.49 (2.12) | 0.62 (0.04) |
| *TEF* | 11.70 (2.40) | 1617.48 (161.22) | 0.7  (0.11) | 67.80 (7.33) | 1.47 (0.18) | 46.20 (2.93) | 0.67 (0.07) |
| *Sham* | 21.34 (10.66) | 1484.42 (86.32) | 1.41  (0.69) | 36.52 (1.90) | 1.43 (0.33) | 41.18 (2.92) | 0.51 (0.05) |
| **35 minutes** |  |  |  |  |  |  |  |
| *TBI+HS* | 19.58 (1.65) | 1797.24 (71.27) | 1.09  (0.08) | 64.26 (2.83) | 1.25 (0.07) | 50.07 (1.58) | 0.61 (0.03) |
| *Mock* | 15.84 (1.70) | 1765.44 (165.72) | 0.9  (0.04) | 61.42 (4.46) | 1.09 (0.09) | 51.67 (2.81) | 0.56 (0.05) |
| *SEF* | 21.94 (3.51) | 1813.18 (80.52) | 1.21  (0.19) | 69.06 (5.78) | 1.25 (0.10) | 52.19 (2.39) | 0.64 (0.04) |
| *TEF* | 20.44 (2.69) | 1808.56 (140.18) | 1.12  (0.10) | 61.88 (4.31) | 1.39 (0.14) | 46.58 (2.84) | 0.63 (0.05) |
| *Sham* | 29.37 (15.45) | 1350.14 (70.22) | 1.98  (0.98) | 41.18 (1.99) | 1.38 (0.34) | 45.14 (3.21) | 0.53 (0.07) |
| **105 minutes** |  |  |  |  |  |  |  |
| *TBI+HS* | 18.22 (1.86) | 1749.76 (73.24) | 1.03  (0.09) | 64.82 (3.04) | 1.35 (0.08) | 45.03 (1.57) | 0.59 (0.03) |
| *Mock* | 14.81 (2.22) | 1695.45 (163.21) | 0.88  (0.11) | 59.27 (4.19) | 1.16 (0.11) | 46.16 (2.84) | 0.53 (0.04) |
| *SEF* | 21.33 (4.15) | 1874.18 (108.01) | 1.12  (0.21) | 70.21 (5.77) | 1.44 (0.15) | 44.95 (3.15) | 0.62 (0.04) |
| *TEF* | 18.05 (2.71) | 1671.90 (117.61) | 1.07  (0.12) | 64.18 (5.35) | 1.42 (0.12) | 44.15 (2.49) | 0.62 (0.04) |
| **210 minutes** |  |  |  |  |  |  |  |
| *TBI+HS* | 14.10 (1.43) | 1709.43 (74.14) | 0.81  (0.06) | 76.03 (4.12) | 1.35 (0.09) | 57.93 (3.04) | 0.75 (0.04) |
| *Mock* | 11.54 (1.39) | 1610.81 (148.13) | 0.73  (0.08) | 72.53 (5.36) | 1.10 (0.11) | 60.05 (4.46) | 0.64 (0.04) |
| *SEF* | 15.94 (2.87) | 1838.86 (96.75) | 0.84  (0.14) | 82.65 (8.66) | 1.41 (0.16) | 60.61 (6.77) | 0.80 (0.07) |
| *TEF* | 14.44 (2.71) | 1664.51 (142.04) | 0.85  (0.11) | 72.41 (6.84) | 1.51 (0.15) | 53.44 (4.29) | 0.78 (0.06) |
| **24 hours** |  |  |  |  |  |  |  |
| *TBI+HS* | 6.68 (0.45) | 1475.90 (78.23) | 0.47  (0.04) | 56.81 (4.30) | 1.27 (0.05) | 51.75 (2.44) | 0.64 (0.02) |
| *Mock* | 8.17 (0.80) | 1390.83 (165.78) | 0.63  (0.10) | 59.25 (4.55) | 1.11 (0.06) | 59.46 (3.06) | 0.66 (0.06) |
| *SEF* | 6.32 (0.76) | 1603.72 (147.32) | 0.41  (0.06) | 59.72 (9.79) | 1.29 (0.06) | 49.47 (3.87) | 0.62 (0.03) |
| *TEF* | 5.97 (0.60) | 1408.84 (103.49) | 0.43  (0.04) | 52.16 (6.27) | 1.37 (0.08) | 48.52 (4.42) | 0.65 (0.04) |
| *Sham* | 10.31 (3.73) | 1315.99 (87.80) | 0.84  (0.32) | 32.98 (2.37) | 1.15 (0.15) | 44.69 (3.52) | 0.47 (0.02) |

*Notes*: Data are presented as mean (standard error of the mean) on the original scale (i.e., not natural log-transformed). KYNA, QUIN, and 3-HK are reported in nmol/L; KYN and TRP are reported in µmol/L. Ratios (KYNA/QUIN and KYN/TRP) are reported as ×10^-2^ for readability.
*Abbreviations*: 3-HK = 3-hydroxykynurenine; HS = hemorrhagic shock; KYN = kynurenine; KYNA = kynurenic acid; QUIN = quinolinic acid; SEF = standard evacuation flight; TBI = traumatic brain injury; TEF = tactical evacuation flight; TRP = tryptophan.
